# Supplementary material for: Mutual information: Measuring nonlinear dependence in longitudinal epidemiological data
Source: PLoS One. 2023 Apr 26;18(4):e0284904. doi: 10.1371/journal.pone.0284904 (PMC10132663; doi:10.1371/journal.pone.0284904)
Supplement: S1 File — (PDF) [file pone.0284904.s001.pdf]

Supplementary Materials to *Mutual information: Mmasuring nonlinear dependence in longitudinal epidemiological data*

Alexander L. Young [1,¶*] Willem van den Boom [2] Rebecca A. Schroeder [3] Vijay Krishnamoorthy [3 &] Karthik Raghunathan [3&] Hau-Tieng Wu [4] David B. Dunson [4,5&]

1 Department of Statistics, Harvard University, Cambridge, Massachusetts, U.S.A.
2 Yong Loo Lin School of Medicine, National University of Singapore, Singapore
3 Department of Anesthesiology, Duke University, Durham, North Carolina, U.S.A.
4 Department of Mathematics, Duke University, Durham, North Carolina, U.S.A.
5 Department of Statistical Science, Duke University, Durham, North Carolina, U.S.A.

* Corresponding Author
E-mail: alexander_young@fas.harvard.edu (ALY)

¶ These authors contributed equally to this work.
& These authors contributed equally to this work.

Contents

1 Entropy and Mutual Information: Definitions 1
1.1 Discrete Distributions 1
1.1.1 Example 2
1.2 Continuous Distributions 3
1.2.1 Example 3

2 Mutual Information: Estimation 3

3 Sampling Error Comparison 4
3.1 Independent Data 5
3.2 Dependent Data 5

4 Effect of k on MI Estimation 7

1 Entropy and Mutual Information: Definitions

1.1 Discrete Distributions

Let X and Y be discrete variables where X can take one of J values $x_1, \ldots, x_J$ and Y can take one of K values $y_1, \ldots, y_K$. Let $p_{jk} = P(X = x_j, Y = y_k)$, $p_j = P(X = x_j)$, and $P(Y = y_k)$ be the joint and marginal probability mass functions (pmfs) of X and Y. The information in an observation having $X = x_j$ is defined to be $-\log p_j$, and similar definitions hold for observations of Y or (X, Y). There is flexibility in the choice of base of the logarithm, so long as it is greater than one. An interpretable choice is base two, which gives information in units of bits. Informally, one bit represents the answer to one yes/no question. In the following exposition and examples, we use base two. For technical reasons, many estimators assume the natural log (giving information in units

Table 1. Probability table for mutual Information example with discrete distributions

	$X = -1$	$X = 0$	$X = 1$	$P(Y = k)$
$Y = 0$	0	1/3	0	1/3
$Y = 1$	1/3	0	1/3	2/3
$P(X = j)$	1/3	1/3	1/3	1

of nats). The change of base formula for logs enables a straightforward rescaling of information estimated in nats to its corresponding value in bits.

We define the entropy of X to be the mean information content

$$H(X) = -\sum_{j=1}^{J} p_j \log_2(p_j).$$

Similar definitions hold for $H(Y)$ and $H(X, Y)$. The mutual information of X and Y is

$$\begin{aligned} I(X,Y) &= \sum_{j=1}^{J}\sum_{k=1}^{K} p_{jk}\log_2\left(\frac{p_{jk}}{p_j p_k}\right) = H(X) + H(Y) - H(X,Y) \\ &= -\sum_{j=1}^{J} p_j \log_2(p_j) - \sum_{k=1}^{K} p_k \log_2(p_k) + \sum_{j=1}^{J}\sum_{k=1}^{K} p_{jk}\log_2(p_{jk}) \end{aligned} \tag{1}$$

One can show $I(X, Y) \geq 0$ and that $I(X, Y) = 0$ if and only if $p_{jk} = p_j p_k$ for all j, k, which is to say when X and Y are independent. The equivalence of independence and zero mutual information is a key property illustrated in the example below.

The average amount of shared information cannot exceed the average amount of information contained in observations of X or Y so that $I(X, Y)$ must be less than both $H(X)$ and $H(Y)$. This natural restriction allows one to define the unitless uncertainty coefficients

$$C_{XY} = \frac{I(X,Y)}{H(Y)}, \qquad C_{YX} = \frac{I(X,Y)}{H(X)},$$

where C_{XY} (C_{YX}) indicates the proportion of information of Y (X) which is shared with X (Y) so that C_{XY} (C_{YX}) is zero if and only if X and Y are independent and one if and only if Y (X) is a deterministic function of X (Y).

1.1.1 Example

Let X be a uniformly distributed random variable with $P(X = j) = 1/3$ for $j = -1, 0, 1$ and let $Y = X^2$. The joint and marginal distributions of X and Y are shown Table 1.

It is straightforward to show that $E[XY] = E[X^3] = 0$ and $Corr(X, Y) = 0$. Conversely, the joint and marginal entropies of X and Y are:

$$\begin{aligned} H(X) &= H(X,Y) = -\sum_{j=-1}^{1} \tfrac{1}{3}\log_2\left(\tfrac{1}{3}\right) = \log_2(3)\, bits, \\ H(Y) &= -\tfrac{1}{3}\log_2\left(\tfrac{1}{3}\right) - \tfrac{2}{3}\log_2\left(\tfrac{2}{3}\right) = \log_2(3) - \tfrac{2}{3}\, bits \end{aligned} \tag{2}$$

so that $I(X, Y) = \log_2(3)–2/3 \approx 0.92$ bits. Note that $H(X)$ and $H(X, Y)$ are equal. Since Y is completely determined by X, an observation of (X, Y) provides no new information beyond the observation of X alone which is reflected in the uncertainty coefficients:

$$C_{XY} = \frac{I(X,Y)}{H(Y)} = 1, \quad C_{YX} = \frac{I(X,Y)}{H(X)} \approx 0.58.$$

The value $C_{XY} = 1$ indicates the deterministic dependence of Y on X.

1.2 Continuous Distributions

When switching from discrete to continuous distributions, it is natural to replace pmfs with pdfs and sums with integrals. Thus, if X and Y are continuous with joint and marginal probability densities, $f(x,y)$, $f_x(x)$, and $f_y(y)$, the mutual information is

$$\begin{aligned} I(X,Y) &= \int_{-\infty}^{\infty}\int_{-\infty}^{\infty} f(x,y)\log_2\left(\frac{f(x,y)}{f_x(x)f_y(y)}\right)dxdy = h(X)+h(Y)-h(X,Y) \\ &= -\int_{-\infty}^{\infty} f_x(x)\log_2(f_x(x))dx - \int_{-\infty}^{\infty} f_y(y)\log_2(f_y(y))dy \\ &\quad + \int_{-\infty}^{\infty}\int_{-\infty}^{\infty} f(x,y)\log_2(f(x,y))dxdy \end{aligned} \tag{3}$$

where $0\log_2 0 \equiv 0$ if $f(x,y)=0$.

Unfortunately, the differential entropies, $h(X)$, $h(Y)$, and $h(X,Y)$, do not carry the same interpretation as their discrete counterparts. An odd but unavoidable challenge with a continuous random variable, X, is that a realization of a X will be an irrational number with probability one. As such, one would require an infinite amount of information to record it. A more detailed discussion of differential entropy can be found in [2]. Despite this limitation, the interpretation of MI remains the same for continuous data as it is a measure of shared information. Importantly, the nonnegativity of MI and its equality to zero if and only if X and Y are independent are both preserved.

Without meaningful definitions of $h(X)$ and $h(Y)$ it is not possible to construct uncertainty coefficients akin to those in the discrete case. This motivates a natural question of how to interpret the units of MI and when it is large. There are no cut-offs for weak, moderate, or strong relationships. Rather, we propose comparing the MI for a pair of covariates relative to the MIs obtained for all other pairs of variables collected in the same study population. This approach is illustrated in the intraoperative HR/MAP application considered in the manuscript. However, we again reiterate that the interpretation of mutual information as shared information between variables is unchanged. In fact, this interpretation extends to mixed data as well.

1.2.1 Example

Let X have a standard Gaussian distribution and given X, suppose that Y is uniformly distributed on the interval $(-|X|-1, |X|-1)$. Similar to the discrete example, one can show that $E[XY]=E[X]=0$ and $Corr(X,Y)=0$. Equation 3 cannot be evaluated directly in this case, but a numerical approximation indicates that $I(X,Y)=2.83$ bits.

2 Mutual Information: Estimation

In principle, one could combine estimates of the joint and marginal entropies to estimate the mutual information. Unfortunately, the pmfs and pdfs involved in these calculations are generally unknown and must be estimated from data. In the case of discrete data, it is natural to replace the pmfs in Equation (1) with observed frequencies. This approach is known to give biased results which have motivated improved estimators [5]. We omit those details and instead focus on the continuous case which is less straightforward but of greater interest to our case study of intraoperative hemodynamic data.

There are a number of density estimation techniques for independent, continuous data such as binning and kernel density estimators. Instead we have elected to focus on a method adapted from a k-nearest neighbor entropy estimation algorithm, which has been developed specifically for mutual information [1]. Important properties of

k-nearest neighbor methods have been shown to hold for time series and longitudinal data, which is of central interest in the study of intraoperative hemodynamic data (Young and Dunson 2019, arXiv:1904.05850).

Consider a collection of N regularly sampled HR and MAP measurements, (HR_i, MAP_i), for $i = 1, \ldots, N$ and fix a positive integer k which is less than N. Let $r_(i, k)$ be the absolute value of the difference between HR_i and its kth closest HR measurement from the set of all other HR measurements $\{HR_{i'}\}$, $i' = 1, \ldots, i-1, i+1, \ldots, N$.

To estimate the HR density at HR_i, we make the following approximations based on Riemann sums

$$2f(HR_i)r_{ik} \approx \frac{e^{\psi(k)}}{N}, \quad -\log f(HR_i) \approx \ln(2Nr_{i,k}) - \psi(k).$$

Here $\psi(k)$ is the digamma function, which is incorporated to reduce bias for small values of k. Importantly, the digamma function assumes use of the natural log resulting in MI and differential entropy estimates in nats, which we assume to be the case hereafter. By the change of base formula, one may simply divide an MI estimate in nats by $\ln(2)$ to obtain the estimate in bits.

For the differential entropy, one can obtain the empirical estimate

$$H(HR) \approx \ln(2N) - \psi(k) + \frac{1}{N}\ln(r_{i,k})$$

and a similar estimate for $H(MAP)$ [3, 4]. The estimate for the joint entropy is

$$H\big((HR, MAP)\big) \approx \ln(4N) - \psi(k) + \frac{2}{N}\sum_{i=1}^{N} \ln\left(r_{i,k}^{HR,MAP}\right).$$

We obtain this estimate by using the maximum absolute difference to determine the ordering of the nearest neighbors of (HR_i, MAP_i). The additional factor of two in the $4N$ and $2/N$ terms appear since (X, Y) is two-dimensional. Combining these estimates together gives the following MI estimator

$$I(HR, MAP) \approx \ln(N) - \psi(k) + \frac{1}{N}\sum_{i=1}^{N} \ln\left(\frac{r_{i,k}^{HR} r_{i,k}^{MAP}}{2\ln r_{i,k}^{HR,MAP}}\right).$$

This estimate can result in biased estimates if the standard deviations in the HR data are drastically different from standard deviations in the MAP data. To address this issue, [1] introduced a bias-corrected version

$$\begin{aligned} I(HR, MAP) \quad &\approx \psi(k) + \psi(N) \\ &-\tfrac{1}{N}\textstyle\sum_{i=1}^{N}\Big(\psi(N_i^{HR}+1) + \psi(N_i^{MAP})\Big) \end{aligned} \tag{4}$$

which we use in this article with $k = 5$ nearest neighbors for the HR/MAP data and $k = 20$ for the gapminder data. Here N_i^{HR} is the number of HR measurements within distance $r_{i,k}^{HR,MAP}/2$ of HR_i and similarly for N_i^{MAP}.

Implementation of this estimator can be found in this github repository.

3 Sampling Error Comparison

Herein, we provide a study of the convergence behavior of three statistics measuring dependence: the Maximal Information Coefficient (MIC) estimated using the *minerva* package [7], distance correlation (dCor) estimated using the *energy* package [6], and MI estimated via nearest neighbors, hereafter referred to as the KSG method. All experiments were conducted in R version 4.2.1.

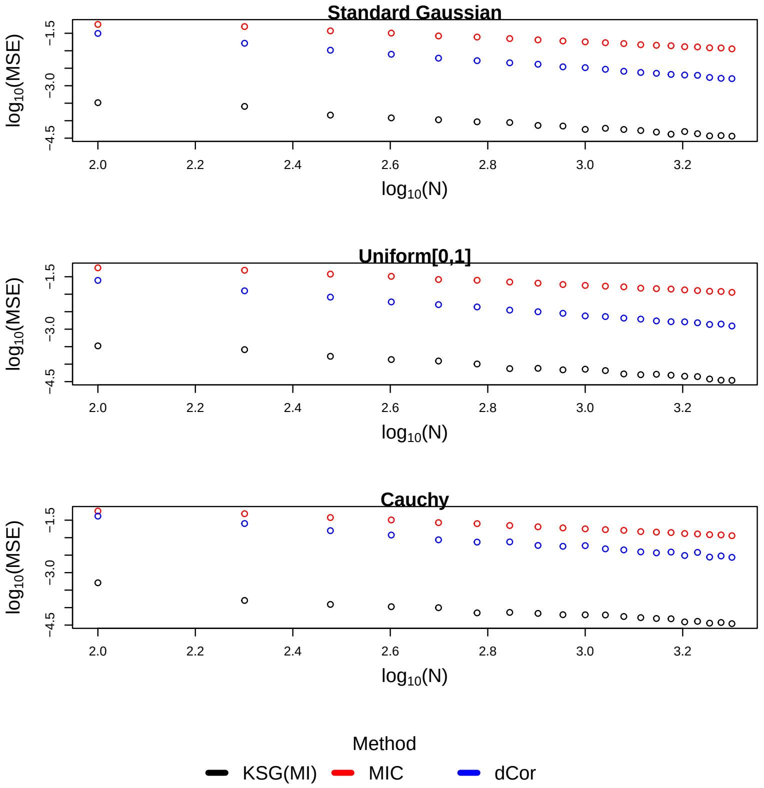

Fig 1. The mean squared error for each method is shown for each sampling distribution. Across all joint distributions of (X, Y) and samples sizes, the KSG estimator of MI (black) has a MSE which is approximately two orders of magnitude smaller than that of MIC (red) or dCor (blue).

3.1 Independent Data

Closed form expressions for MI, MIC, and dCor for a given joint distribution are, in general, intractable with a few exceptions, such as a bivariate Gaussian distribution in the case of MI and dCor. Thus, empirical studies on the mean squared error of statistical estimators of these quantities are somewhat limited. Thus, we will begin with the case of independent variables wherein each measure is zero.

We consider three cases for *iid* random variables X and Y: $N(0,1)$, $Unif[0,1]$, and Cauchy. In each case, 400 independent trials of N *iid* draws of (X, Y) are generated. For each trial, MIC, dCor, and MI are estimated from the N samples of (X, Y). The mean squared error (MSE) from zero of the MI, MIC, and dCor methods are then estimated from these results. The process is repeated for $N = 100, 200, \ldots, 2000$ samples. Figure 1, provides graphs of the MSE as a function of sample size, N.

In each case, the KSG estimator has MSE approximately two orders of magnitude smaller than that of MIC or dCor across all samples size considered. Notably, KSG outperforms either method over the range of HR and MAP samples considered in the main article, thereby supporting the choice of MI as a measure of statistical dependence.

3.2 Dependent Data

It is natural to wonder if the results of the preceding example hold in the case of dependent data. Herein, we consider the case where random variables X, Y follow a

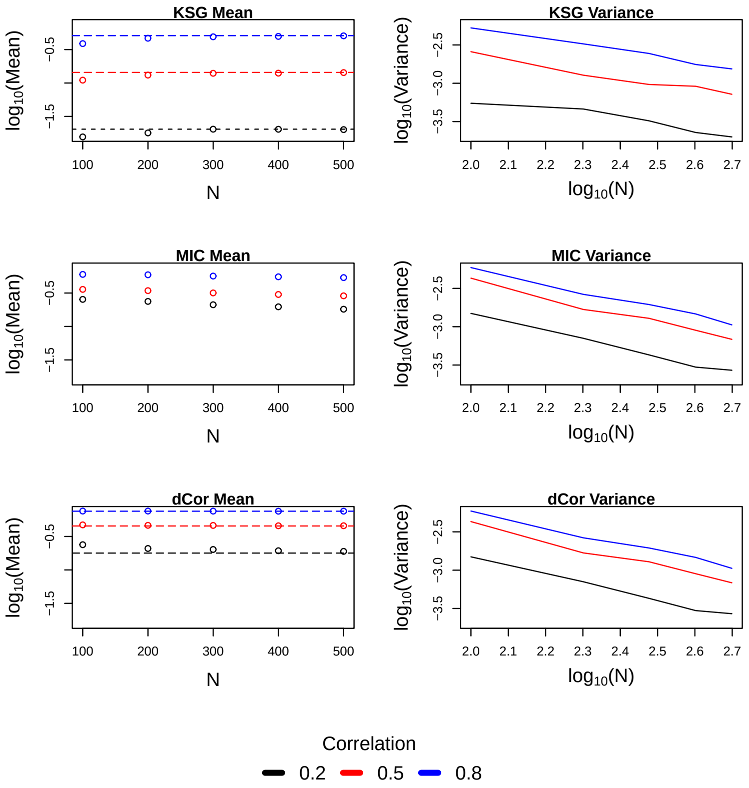

Fig 2. The sample means and variances of each statistic are shown as a function of sample size for the values $\rho = 0.2$(black), 0.5(red), and 0.8(blue) . For MI and dCor, true values are shown as dashed lines with the corresponding color.

bivariate Gaussian distribution with mean $\mu = (0, 0)$ and covariance matrix

$$\Sigma = \begin{bmatrix} 1 & \rho \\ \rho & 1 \end{bmatrix}.$$

In this case,

$$I(X, Y) = -\frac{1}{2}\log(1 - \rho^2) \text{ nats}$$
$$dCor(X, Y) = \frac{\rho \arcsin(\rho) + \sqrt{1 - \rho^2} - \rho \arcsin(\rho/2) + \sqrt{4 - \rho^2} + 1}{1 + \pi/3 - \sqrt{3}}.$$

MIC does not have a known value in this setting.

We consider three cases: $\rho = 0.2, 0.5, 0.8$. For each value of ρ, we generate N samples of (X, Y) and estimate MI, MIC, and dCor. Using 400 independent realizations of this process, empirical estimates for the mean and variance of each dependence measure are calculated. This process is repeated for samples sizes $N = 100, 200, \ldots, 500$. The results are shown in Figure 2.

All methods exhibit comparable variances across each distribution and sample size, One exception is KSG for $\rho = 0.2$ which has a much lower variance for small sample sizes. However, the results pertaining to the mean (and thus sample bias) are striking. dCor and KSG have comparable bias across all sample sizes and correlation. Notably, the bias is neglible when compared with the true value for $N \geq 300$. However, the

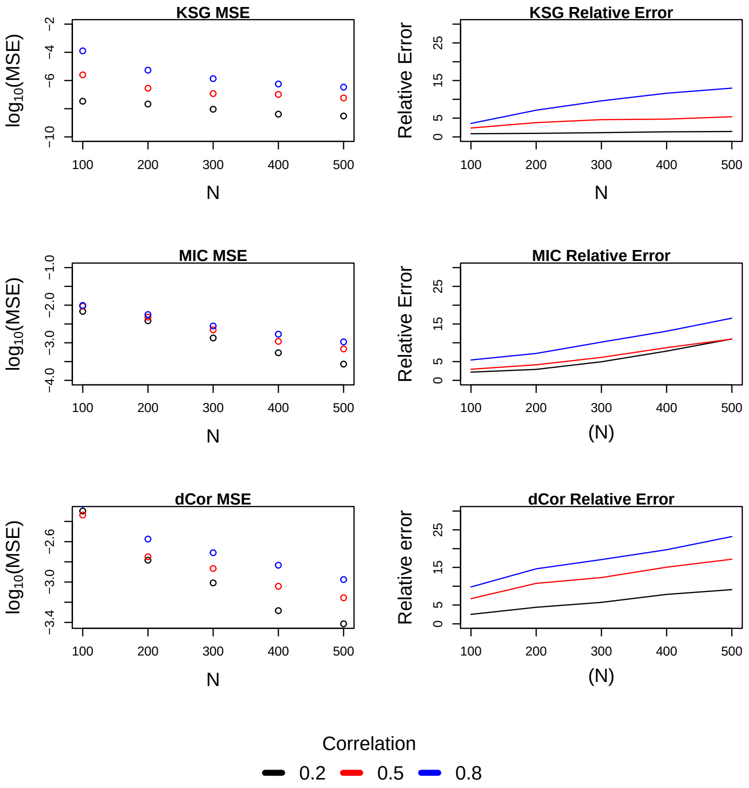

Fig 3. The MSE and Relative Error of each statistic are shown as a function of sample size for the values $\rho = 0.2$(black), 0.5(red), and 0.8(blue) . For MIC, we use the estimated value for the largest sample size as the true value.

sample mean MIC is clearly decreasing for all values of N suggesting that the finite sample bias of MIC decays much more slowly than KSG or dCor.

Based on the preceding results, one may expect that KSG will have lower mean squared error (MSE) when compared to MIC and dCor in this setting as well. However, lower MSE may be an artifact of smaller overall values of an estimand. For comparison, we also provide plots for MSE and relative error, as measured by the ratio of the bias and root mean squared error (RMSE) for each method and correlation. These summaries are shown in Fig. 3. When estimating the bias, true values for KSG and dCor were available. However, MIC has no known ground truth and was instead estimated using the mean results for the highest sample sizes. Interestingly, in all cases the relative error was an increasing function of sample size, which is a result of the bias decreasing at a slower rate than the RMSE. Importantly, KSG has the lowest relative error in all correlations and sample sizes considered.

Collectively, these results indicate that MI, as estimated using KSG, outperforms the other methods consider across a range of test scenarios when one is most concerned about robust estimation under moderate sample sizes. As such, the choice of MI is a suitable measure of dependence in the primary study of HR and MAP.

4 Effect of k on MI Estimation

Optimal tuning of the number of nearest neighbors for the KSG estimator is unknown. Below, we show the MI estimates of the gapminder data using $k = 10$ to demonstrate

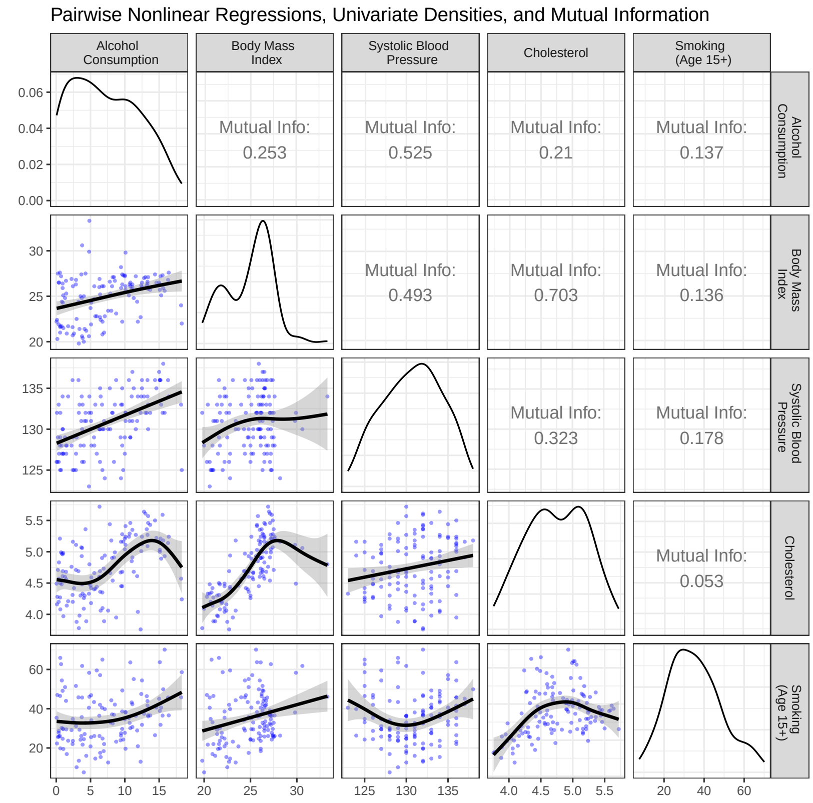

Fig 4. MI estimates using the KSG estimator with $k = 10$.

the impact of this choice. For reference, the $k = 20$ case considered in the paper suggests SBP and cholesterol have the strongest association, followed by BMI and cholesterol, and SBP with alcohol consumption and BMI. For $k = 10$, the four strongest pairwise associations are the same, but cholesterol and BMI is the weakest in this group.

References

1. Kraskov A, Stögbauer H, Grassberger P. Estimating Mutual Information. Phys Rev E. 2004;69(6):66138. doi:10.1103/PhysRevE.69.066138.

2. Cover TM, Thomas JA. Elements of information theory. 2nd ed. John Wiley & Sons, Inc.; 2006.

3. Berrett TB, Samworth RJ, Yuan M. Efficient multivariate entropy estimation via k-nearest neighbour distances. Ann Stat. 2019;47(1):288–318. doi:10.1214/18-AOS1688.

4. Delattre S, Fournier N. On the Kozachenko–Leonenko entropy estimator. J Stat Plan Inference. 2017;185:69–93. doi:10.1016/j.jspi.2017.01.004.

5. Schürmann T. Bias analysis in entropy estimation. Journal of Physics A: Mathematical and General. 2004;37(27):L295.

6. Rizzo M, Székely G. energy: E-Statistics: Multivariate Inference via the Energy of Data. 2022, R package version 1.7-10,

7. Albanese D et al. Minerva and minepy: a C engine for the MINE suite and its R, Python and MATLAB wrappers. Bioinformatics. 2012.
