# Supplementary material for: Mutual information: Measuring nonlinear dependence in longitudinal epidemiological data
Source: PLoS One. 2023 Apr 26;18(4):e0284904. doi: 10.1371/journal.pone.0284904 (PMC10132663; doi:10.1371/journal.pone.0284904)
Supplement: Attachment — Submitted filename: Response to Reviewers.docx [file pone.0284904.s002.docx]

Alexander L. Young, PhD

Department of Statistics

Harvard University

Science Center 400 Suite

One Oxford St

Cambridge, MA 02138

[alexander_young@fas.harvard.edu](mailto:alexander_young@fas.harvard.edu)

## Eugene Demidenko, Ph.D.

Academic. Editor

*PLOS One*

## Dear Dr. Demidenko,

Below, please find are responses to the issues identified in our initial submission of *Mutual information: measuring nonlinear dependence in longitudinal epidemiological data*. Prior to those details, please let me apologize for the delay in response. De-identifying and preparing our patient data took considerably more time than we expected, but we have been able to make it available with publication.

**Reviewer Comments**

1. *The problem of using mutual information to discover nonlinearities is well addressed in the literature. The so called 'maximal information coefficient,' or MIC, is introduced in
   Kinney, J.B. and Gurinder S. A. (2014) Equitability, mutual information, and the maximal information coefficient, PNAS 111:119
   More discussion can be found in
   David N. Reshef, Yakir A. Reshef, Hilary K. Finucane, Sharon R. Grossman, Gilean McVean, Peter J. Turnbaugh, Eric S. Lander, Michael Mitzenmacher, Pardis C. Sabeti (2011). Detecting Novel Associations in Large Data Sets, Science 334.
   Moreover, there is an R package that allows computation of MIC, minerva.
   The authors miss these critical references.*

We thank the reviewer for supplying these valuable references which have been included in the revised version of the manuscript.

2. *The paper is poorly written. For example, the Appendix is full of?*

The writing has been revised in several areas throughout the manuscript with a small reordering of the material to streamline the exposition. The multiple occurrences of ?? in the supplementary material resulted from an issue with BibTex and has been addressed in the updated version.

**Journal Requirements**

1. *Please ensure that your manuscript meets PLOS ONE's style requirements, including those for file naming. The PLOS ONE style templates can be found at*

   The author affliation symbols have been updated in concordance with the journal guidelines. Figure and Table citations have been arranged accordingly as well. The PLoS One style package was used to generate the manuscript.

*2. Please include a complete ethics statement in the Methods section, including the name of the IRB and the approval number, and whether they approved the study or waived the need for approval.*

The following ethics statement has been added to the Methods Section:

The Duke Medicine Institutional Review Board for Clinical Investigations approved this study (approval no.: Pro0067683). After full review, the requirement for informed consent of this retrospective study was waived as the data were analyzed anonymously.

*3. Thank you for stating the following financial disclosure:*

*“ALY was supported by the National Science Foundation (Award #1045153, Award 331 #1546130)*

*WvdB was supported by the National Institute of Environmental Health 332 Sciences (NIEHS) of the National Institutes of Health (NIH) (R01-ES017240)”*

*Please state what role the funders took in the study.  If the funders had no role, please state: "The funders had no role in study design, data collection and analysis, decision to publish, or preparation of the manuscript."*

*If this statement is not correct you must amend it as needed.*

*Please include this amended Role of Funder statement in your cover letter; we will change the online submission form on your behalf.*

Here is our updated financial disclosure which has been added to the updated cover letter as well.

ALY was supported by the National Science Foundation (Award #1045153, Award 331 #1546130). WvdB was supported by the National Institute of Environmental Health 332 Sciences (NIEHS) of the National Institutes of Health (NIH) (R01-ES017240). The funders had no role in study design, data collection and analysis, decision to publish, or preparation of the manuscript.

*4. Thank you for stating the following in the Acknowledgments Section of your manuscript:*

*“ALY was supported by the National Science Foundation (Award #1045153, Award #1546130). WvdB was supported by the National Institute of Environmental Health Sciences (NIEHS) of the National Institutes of Health (NIH) (R01-ES017240). The funders had no role in study design, data collection and analysis, decision to publish, or preparation of the manuscript.”*

*We note that you have provided additional information within the Acknowledgements Section that is not currently declared in your Funding Statement. Please note that funding information should not appear in the Acknowledgments section or other areas of your manuscript. We will only publish funding information present in the Funding Statement section of the online submission form.*

*Please remove any funding-related text from the manuscript and let us know how you would like to update your Funding Statement. Currently, your Funding Statement reads as follows:*

*“ALY was supported by the National Science Foundation (Award #1045153, Award 331 #1546130)*

*WvdB was supported by the National Institute of Environmental Health 332 Sciences (NIEHS) of the National Institutes of Health (NIH) (R01-ES017240)”*

*Please include your amended statements within your cover letter; we will change the online submission form on your behalf.*

We have removed the acknowledgments section from the manuscript. Please see item 4 above regarding our amended funding statement.

*5. In your Data Availability statement, you have not specified where the minimal data set underlying the results described in your manuscript can be found. PLOS defines a study's minimal data set as the underlying data used to reach the conclusions drawn in the manuscript and any additional data required to replicate the reported study findings in their entirety. All PLOS journals require that the minimal data set be made fully available. For more information about our data policy, please see* [*http://journals.plos.org/plosone/s/data-availability*](http://journals.plos.org/plosone/s/data-availability)*.*

*Upon re-submitting your revised manuscript, please upload your study’s minimal underlying data set as either Supporting Information files or to a stable, public repository and include the relevant URLs, DOIs, or accession numbers within your revised cover letter. For a list of acceptable repositories, please see* [*http://journals.plos.org/plosone/s/data-availability#loc-recommended-repositories*](http://journals.plos.org/plosone/s/data-availability#loc-recommended-repositories)*. Any potentially identifying patient information must be fully anonymized.*

*Important: If there are ethical or legal restrictions to sharing your data publicly, please explain these restrictions in detail. Please see our guidelines for more information on what we consider unacceptable restrictions to publicly sharing data:* [*http://journals.plos.org/plosone/s/data-availability#loc-unacceptable-data-access-restrictions*](http://journals.plos.org/plosone/s/data-availability#loc-unacceptable-data-access-restrictions)*. Note that it is not acceptable for the authors to be the sole named individuals responsible for ensuring data access.*

*We will update your Data Availability statement to reflect the information you provide in your cover letter.*	Here is our updated Data Availability Statement which has been added to the manuscript:

The data used for this study has been anonymized and made available through the Duke University Library Digital Repository for Research Data, doi:10.7924/r45q52g2t.

*6. Please include captions for your Supporting Information files at the end of your manuscript, and update any in-text citations to match accordingly. Please see our Supporting Information guidelines for more information:* [*http://journals.plos.org/plosone/s/supporting-information*](http://journals.plos.org/plosone/s/supporting-information)*.*

A caption for the Supporting Information files has been added to the end of the manuscript.

Thank you again for considering our manuscript. We will be happy to address any additional changes or updates you require.

Sincerely,

Alexander L Young

Department of Statistics

Harvard University
