# Supplementary material for: Mutual information: Measuring nonlinear dependence in longitudinal epidemiological data
Source: PLoS One. 2023 Apr 26;18(4):e0284904. doi: 10.1371/journal.pone.0284904 (PMC10132663; doi:10.1371/journal.pone.0284904)
Supplement: Attachment — Submitted filename: Response to Reviewers.docx [file pone.0284904.s003.docx]

Alexander L. Young, PhD

Department of Statistics

Harvard University

Science Center 400 Suite

One Oxford St

Cambridge, MA 02138

[alexander_young@fas.harvard.edu](mailto:alexander_young@fas.harvard.edu)

## Eugene Demidenko, Ph.D.

Academic. Editor

*PLOS One*

## Dear Dr. Demidenko,

Below, please find our responses to the issues identified in our revised submission of *Mutual information: measuring nonlinear dependence in longitudinal epidemiological data*. We thank you for the opportunity to provide additional results supporting our choice of Mutual Information over competing methods. Furthermore, we agree that the additional details do strengthen the paper.

**Reviewer Comments**

*1. You did not address my concern on the existing method of estimation of mutual information via MIC. It is not sufficient to cite the MIC research papers. More discussion and comparison must be provided. What if your estimator is equivalent to or even worse than MIC?*

This is an excellent point, and we appreciated the opportunity to address it. We have expanded the discussion on estimators of statistical dependence including references to distance correlation. During this discussion, we note the emphasis on power in this related literature is of less import than minimizing error when estimating quantities from finite samples. We address this issue through an empirical study on mean squared error included in the supporting materials.

*2. The way to compare via simulations is to pick a distribution, say a multivariate normal distribution, and show how the MSE of both estimators converge to the theoretical (closed form) counterpart as a function of the number of observations.*

We agree with this point. However, aside from mutual information and distance correlation in a few analytically tractable cases, estimators of statistical dependence do not have closed form expressions. As such, we have included two separate empirical studies.

In the first, we focus on independent data in three settings (Gaussian, uniform, and Cauchy distributions) where mutual information, MIC, and distance correlation are all zero. These results suggest that MI as estimated using the nearest neighbor approach has mean squared error approximately two orders of magnitude lower than MIC or distance correlation across all sample sizes considered.

In the second, we consider the bivariate Gaussian case with non-zero correlation. In this setting, both mutual information and distance correlation have closed form expressions. MIC does not. Thus, instead of focusing on MSE, we provide empirical estimates of the mean and variance of each estimator for comparison. All methods exhibit comparable variance across the chosen sample sizes. When studying the empirical means, the fluctuations around the true values of MI and dCor are comparable indicating similar small sample bias whereas the sample mean of MIC does not appear to have converged by the maximum sample size considered.

These studies, which we believe provide compelling justification for our methodology, were included in the supporting materials. We are open to moving these results into the main paper pending input from the reviewers.

Thank you again for considering our manuscript. We will be happy to address any additional changes or updates you require.

Sincerely,

Alexander L Young

Department of Statistics

Harvard University
