# Supplementary material for: Mutual information: Measuring nonlinear dependence in longitudinal epidemiological data
Source: PLoS One. 2023 Apr 26;18(4):e0284904. doi: 10.1371/journal.pone.0284904 (PMC10132663; doi:10.1371/journal.pone.0284904)
Supplement: Attachment — Submitted filename: Response to Reviewers Round 2.docx [file pone.0284904.s004.docx]

Alexander L. Young, PhD

Department of Statistics

Harvard University

Science Center 400 Suite

One Oxford St

Cambridge, MA 02138

[alexander_young@fas.harvard.edu](mailto:alexander_young@fas.harvard.edu)

## Eugene Demidenko, Ph.D.

Academic. Editor

*PLOS One*

## Dear Dr. Demidenko,

We thank you for considering our manuscript and for the additional detailed comments of your reviewers. Their comments would, indeed, strengthen the results for the perioperative risk assessment community. However, our focus in this article is to demystify the use of mutual information rather than providing optimal risk assessment tools. We have made comments throughout the manuscript to clarify this point and to address some of the additional remarks regarding clarity and typographical issues. Detailed comments to each point are provided below.

**Reviewer 1 Comments**

1. *The authors should justify their need to resort to the logistic regression technique as a baseline model for data analysis and why not other models, especially gradient-boosting decision trees and/or neural networks. A comparative analysis with at least decision trees is much appreciated.*

   We greatly appreciate this comment. A broad study of the different modeling frameworks for mortality prediction is clearly of interest to the clinical care community. However, our aim in this article is to introduce and demystify Mutual Information. As such, we have elected to focus on building intuition and interpretability of MI. The examples were chosen to demonstrate improvements to existing models which are attainable with the inclusion of nonlinear dependence as measured by MI.
2. *Comparative experimental results among Pearson correlation and other robust correlation measures such as distance correlation, mutual information, and maximal information coefficient should be depicted to support the hypothesis.*

   The absolute value of the Pearson correlation is included already. The use of signed Pearson correlation does not alter the results, and we have added a comment on this issue to the manuscript. For the remaining methods, we would like to reemphasize the analysis in the supplemental materials on the estimation of MI vs other methods. Additional details on that analysis are discussed below.

3. *Can the ratios and higher-order statistic relations (square, cubic, etc) in terms of the vital signs among the data be useful to be considered? What are the advantages and disadvantages?*

   Standard deviation of Heart Rate and Mean Arterial Pressure were included. By using generalized additive models, we have thus included quadratic statistics, though we admit that these results are not clearly interpretable from the presented graphs. Ratios and higher-order terms were not considered. We agree that one could consider such terms as a method of improving model fit at the cost of model complexity and the risk of overfitting. However, as we discussed in the introduction on R^2^, the inclusion of such terms is often ad hoc. We have expanded on this observation in the introduction.
4. *The discussion section can highlight the related studies used to explore the statistical dependencies among the vital signs for mortality risk assessment. The reviewer recommends the authors consult and cite the below-mentioned references. The most important must also be discussed, not just mentioned in a list of references.*

   We greatly appreciate the recommend references. In particular, the sequence of studies by Neseragi et al are of central interest to this article as they provide an additional example motivating the use of MI over correlation and ratio-based statistics. We have added detail on these results to our introduction.
5. *Authors must enhance their experiments and try to present results based on evidence. Reporting AUROC, and MSE as performance metrics for mortality risk prediction is not fully correct. Reporting AUPRC, and PPV might add clinical significance. Even, further analysis of the variation of β on Fβ measure can serve the purpose to some extent in terms of robustness and significance.*

   Again, we would like to emphasize that the motivation of the article is to introduce MI and its utility to the perioperative risk assessment community, not to identify optimal models. As such, we believe keeping the model assessment parsimonious to emphasize the interpretation of MI earlier in the article is optimal.

**Reviewer 2 Comments**

On MI:

1. *I agree that R^2 is not a good measure if relationships are highly nonlinear. As long as the relation is monotone, a rank-correlation can be used instead. It is certainly useful to look for better and more general measures. The authors propose to use MI, which quantifies association more robustly. They give an example with the gapminder data. For the relation between SBP and cholesterol, MI is clearly better than R^2. However, I find it difficult to believe that MI is larger than for BMI and cholesterol, which show a much stronger association in the scatterplot. This suggests that R^2 is a better measure in case the association is fairly linear. I would like to see some discussion on this by the authors.*

   This raises a very interesting point on the dependency of tuning k in the nearest neighbor method. Choosing k=10 results in a relationship which is consistent with the reviewers assessment. We have discussed this sensitivity to parameter tuning as a weakness of MI estimation and provided complete result for the gapminder data using k=10 in the supplementary material.
2. *In the supplementary material, three statistics are compared using MSE. I am not convinced that MI is better. MI, MIC and cCor are different quantities. Even if they are standardized, a value of say 0.1 can have a different meaning for each of them. There is no standardized way to compare them.

   This is also seen in Figure 2, where data with correlation is compared. Estimates agree fairly well with true values, except maybe for N=100. KSG has smaller values, but that doesn't mean it gives a better reflection of correlation. I don't see a justification for the claim "one expects KSG to have lower MSE when compared to MIC and dCor in this setting as well". The lower variance for KSG is to be expected given the lower average value.*

   This is an excellent point. We have expanded the analysis in the supplement to also compute relative error, as measured by the ratio of bias and root mean squared error. In all cases considered, KSG has the lowest relative error.

   On the data set:
1. *Do the authors expect that the relation between hemodynamic variables and outcome remains the same if HR and MAP are modified via an intervention?*

   MAP (and HR in some hospitals) are already modified by an intervention. How one could intervene on I(HR,MAP) is unknown at present.
2. *The authors consider a Cox (CPH) model and a logistic regression (LR). For the LR, the model including hemodynamic variables improves with respect to AUC (Table 4). Figure 5 shows two individuals; for the second one, MI is clearly better than R^2. However, the authors do not report to what extent MI improve upon HR and MAP itself with respect to AUC. Also, I would like to see the AUC for the CPH model included.*

   Again, we would like to maintain a focus on MI and the interesting connection between decreased dependence and mortality risk rather than model validation.
3. *Several selections were made. Why were those with ASA score 5 excluded? In Table 2 I see that the number with ASA 1 or 2 is even smaller, while these individuals were not excluded.*

   ASA 5 patients are critically ill and require surgical intervention. We removed those cases from the analysis to focus on settings where surgical status was not dictated by an immediate medical need. We have added a statement on this issue to the article. *Excluding those with fewer than 240 HR/MAP values gives a risk of selection bias. It may be that these individuals have shorter follow-up due to death. The authors include the number of measurements as additional variable (in CPH only), but I don't see these results reported.*

   Hazard ratios for the number of measurements in each surgery type have been added with a brief comparison of these results with the existing discussion on hazard ratios.
4. *Were there any right censored individuals within 30 days?*

   All cases where the patient survived for more than one year were censored.
5. *Correlation does show strong relation with mortality in Fig 6D, but the authors write " does not display a strong association with mortality"*

   This is a fair point. We have updated the language to note association of correlation with outcome in the cardiac cases.
6. *What is the biological reason to include MI in the model? Why would MI relate to mortality?*

   The dependence of HR and MAP has been considered in other studies. In particular, the rate pressure product (RPP) has been considered in other articles including the Krishnamoorthy et al. article in our references. When discussing other means of measuring this association between mixed second moments, the mathematical side of our research team suggested MI given issues on nonlinear behavior of RPP. At present, tt is unclear how MI may relate to mortality through a clear biological mechanism, and we can only offer circumspection which is not justifiable to include in the article. Nonetheless, we do believe this finding could be of interest and have added a comment to the manuscript.
7. *If two variables have a clear trend in individuals over time, it may be better first to remove that trend. It will give a strong correlation, which may have little biological relevance. It may be better to quantify the correlation after removal of the trend.*

   This is a natural point, but removing a trend could leave a nonlinear association in the residuals which would be missed by correlation.

   On Supplementary material:
1. *0 log 0 is zero mathematically, it is not a convention.*

   Informed minds can disagree on the technicalities of this point. The function x log(x) is undefined at x=0, hence our point on the convention of taking 0log(0) = 0, which is a common remark in the information theoretic literature. However, the limit as of x log(x) is zero as x approaches zero and it is thus natural to take 0 log(0) = 0 and treat it as a definition. These details do not add substance to the discussion, and we have elected to remove the language regarding convention in line with the reviewer's remarks.
2. *I don't understand why the entropies for continuous distributions do not carry the same amount of information and why " an infinite number of entries after the decimal" is a problem. They are integrals that can be approximated as accurately as on wishes (e.g. via Riemann sums).*

   This is an excellent question, and the Cover text, which we have cited in the supplement, provides a complete answer.

   Briefly, we will provide some insight here. As a counterpoint, first consider the discrete case first for a finite set {1,…,N} of possible outcomes with associated probabilities p_1_, …, p_N_. The entropy, as measured in bits, provides information on the average number of yes/no answers one needs answered on average to identify a random sample from {1,…,N} based on the probabilities. If p_j_=1 for some j, the entropy is zero since the outcome would be known a priori. If the distribution is uniform, log_2_(N) yes/no questions are needed to identify the outcome, which is obtainable by splitting the possible outcomes into approximately even sized disjoint sets and querying if the realization is in one of these sets. One can the proceed sequentially to finer subsets until the realization is identified.

   If the outcome space is continuous, the potential realizations of the random outcome are irrational numbers with probability 1, hence our comment about the infinite number of entries after the decimal. One could elect to discretize the continuous distribution into partitions at which point identifying which partition contains the sample is almost equivalent to the discrete case above. The corresponding Riemann sum approximation to the integral provides the associated entropy of the discretized case minus an additional term of the form log (partition size) which accounts for the discretization error. As one sends the partition size to 0, the log (partition size) term is formally dropped leaving the differential entropy. If one were to keep this term the entropy discretized approximation would diverge to infinity as the partition size shrunk to zero.

   We have attempted to improve this explanation by noting that the outcome must be irrational, hence impossible to store in finite memory. Additional details on the delicate nature of this point are directed to the reference by Cover et al.
3. *How is k chosen?*

   k was chosen for to balance faster estimation with minimal sampling variability as data were jittered. We have added this point to the manuscript.

Thank you again for considering our manuscript. We will be happy to address any additional changes or updates you require.

Sincerely,

Alexander L Young

Department of Statistics

Harvard University
